# A meta-analysis of the effects of vitamin C supplementation for pregnant smokers on the pulmonary function of their offspring

**DOI:** 10.1186/s12884-024-06377-3

**Published:** 2024-03-07

**Authors:** Lei Wang, Lina Wei, Zhongtian Wang, Xiaoting Ren, Fushuang Yang, Liping Sun

**Affiliations:** 1 School of Chinese Medicine, Changchun University of Chinese Medicine, Changchun Jilin, 130117 China; 2 Pediatric Department, The Affiliated Hospital of Changchun University of Chinese Medicine, Changchun Jilin, 130021 China; 3 Children’s Diagnosis and Treatment Center, Affiliated Hospital of Changchun University of Chinese Medicine, No. 185, Shenzhen Street, Erdao District, Changchun, Jilin China

**Keywords:** Vitamin C, Pulmonary function, Pregnant, Smoke

## Abstract

**Background:**

At present, the need for vitamin C supplementation for pregnant smokers has not been fully studied. This study is aimed at investigating whether vitamin C supplementation for pregnant smoking women can improve the pulmonary function of their offspring.

**Methods:**

Four databases were searched from inception to April 1, 2023 for studies on the effect of vitamin C supplementation to pregnant smokers on the pulmonary function of their offspring. Meanwhile, the reference lists of relevant studies were manually searched. The risk of bias in the included studies was assessed using the Cochrane Collaboration tool, and the data was analyzed using STATA/SE 17.0.

**Results:**

Four randomized controlled trials (RCTs), all of high quality, were enrolled in this meta-analysis, including 787 pregnant women. The offspring of pregnant smokers who received vitamin C supplementation exhibited improved Forced Expiratory Flow between 25 and 75% (FEF25-75), FEF50, FEF75, and Forced Vital Capacity (FVC) compared to those who did not receive vitamin C supplementation. However, there was no statistically significant difference in Forced Expiratory Volume at 0.5 s (FEV0.5) and the ratio of FEV0.5 to FVC between the offspring of pregnant smokers who received vitamin C and the control group.

**Conclusion:**

Vitamin C supplementation for smoking pregnant women may enhance the pulmonary function of their offspring, particularly in FEF25-75, FEF50, FEF75, and FVC. Nevertheless, there are no significant differences in FEV0.5 and the FEV0.5/FVC ratio. These findings suggest that vitamin C supplementation has potential benefits for specific pulmonary function. Further studies are needed to comprehensively assess the effects of vitamin C on pulmonary function in the context of maternal smoking during pregnancy.

**Supplementary Information:**

The online version contains supplementary material available at 10.1186/s12884-024-06377-3.

## Background

Tobacco use is widespread among pregnant women on a global scale. In Europe, 8.1% of pregnant women smoke, while in the United States, the rate is 5.9% [1]. Studies have consistently reported that the prevalence of smoking among pregnant women is positively correlated with the economic development in the region [2]. Smoking during pregnancy poses a threat to both mothers and fetuses. Studies have demonstrated that maternal smoking during pregnancy can increase the risk of low birth weight [3] and congenital defects in fetuses [4], along with long-lasting consequences on the pulmonary function of their offspring. Prenatal exposure to tobacco smoke may elevate the likelihood of asthma and wheezing in offspring [5]. Lung development in the fetus begins in the uterus during the first trimester of pregnancy [6]. Nicotine can pass from the maternal bloodstream through the placenta and enter the fetus, thereby affecting fetal lung function [7]. A case-control study that enrolled 196 pregnant women with ectopic pregnancies reported that the odds ratio (OR) for ectopic pregnancy increased with the number of cigarettes smoked per day. The OR was increased to 3.5 for those who smoked over 20 cigarettes per day [8]. Shobeiri et al. revealed an increased likelihood of placental abruption [9] and a higher vulnerability to miscarriage among pregnant smokers [10]. Findings from animal experiments indicated that nicotine exposure caused airway stenosis or thickening of airway walls [11]. Therefore, it is essential to implement early intervention for pregnant smokers.

Vitamin C is a reducing agent that provides an electron to the substrate and oxidizes itself to ascorbic free radicals, which are relatively stable. Two molecules of ascorbic free radicals can differentiate into one molecule of ascorbic acid and one molecule of dehydroascorbic acid, representing the fully reduced and oxidized forms of vitamin C, respectively. Hence, vitamin C is also known as ascorbic acid. Initially isolated by Albert Szent Gyorgy in 1928, vitamin C was confirmed to be a water-soluble anticoagulant factor in 1932 [12]. With a relatively simple structure and small molecular weight, vitamin C is abundant in various fruits and vegetables [13]. An animal experiment demonstrated that vitamin C supplementation to nicotine-exposed rhesus monkeys during gestation effectively counteracted the adverse effects of nicotine on expiratory flow [14]. Leah et al. discovered that vitamin C supplementation for pregnant smokers during re-pregnancy effectively reduced the risk of asthma and asthma-related mortality in the offspring [15]. However, some have questioned the efficacy of vitamin C supplementation for pregnant smokers, especially when these individuals remain unable to quit smoking despite receiving treatment from a scientific team [13]. Smoking cessation is widely considered the most critical intervention during pregnancy, as it outperforms vitamin C supplementation for pregnant smokers by effectively reducing the intake of combustion by-products, such as nicotine, tar, and carbon monoxide [16]. Consequently, we conducted a meta-analysis to investigate the association between vitamin C supplementation during pregnancy and pulmonary function in offspring.

## Methods

This study followed the Cochrane Handbook for Systematic Reviews of Interventions. This meta-analysis was performed according to the Preferred Reporting Items for Systematic Reviews and Meta-analyses (PRISMA) guidelines [17]. It was registered with the PROSPERO International Prospective Registry for Systematic Reviews (PROSPERO registry number: CRD42023421579) [18].

### Data sources and searches

All studies related to vitamin C supplementation for pregnant smokers were searched in PubMed, Cochrane Library, Web of Science, and Embase databases up to April 2023. Additionally, their reference lists were screened to ensure that no studies were missed. There were no language restrictions on the search. Relevant MeSH keywords were utilized in both Persian and English databases, including ‘Acid, Ascorbic’, ‘Acid, L-Ascorbic’, ‘Ascorbate, Sodium’, ‘Ascorbic Acid’, ‘Magnorbin’, ‘Vitamin C’, ‘Ascorbate, Ferrous’, ‘Ascorbic Acid, Monosodium Salt’, ‘Magnesium Ascorbate’, ‘Pregnant Women’, ‘pregnant woman’, ‘Women, Pregnant’, ‘Woman, Pregnant,’ with their combinations using the operators ‘OR’ and ‘AND.’ The detailed search strategy is provided in Supplementary Material 1.

Studies were included if they met the following inclusion criteria:

1. The studies focused only on pregnant women aged over 18 years who smoked; (Smoking pregnant women included in this study should smok at least 1 ciggerate per day.)
2. Vitamin C was supplemented during pregnancy;
3. The indicators of pulmonary function in the offspring were reported;
4. The study design was randomized controlled trials (RCTs).

Studies were excluded for the following reasons:

1. Participants were pre-diagnosed with asthma, emphysema, and cancer;
2. Participants with lung injury;
3. Case reports, animal and cell studies, reviews and meta-analyses, and conference abstracts;

### Data extraction and quality assessment

Data were extracted by two researchers. For each study included, the extracted information included the author’s name, location, year of publication, study design, country of study, subjects studied, age of pregnant women, BMI, smoking status, and pulmonary function status of the offspring. The retrieved studies were screened by two researchers independently, and any disagreements were resolved by discussion.

The risk of bias was assessed using the Risk of Bias Assessment Tool 2.0 (ROB 2.0), as recommended by the Cochrane Handbook [19]. ROB 2.0 comprises five primary domains: bias arising from randomization, bias due to deviations from established interventions, bias from missing data on outcomes, bias in outcome measurement, and bias in the selective reporting of outcomes. The studies were categorized as “low risk of bias”, “some concerns”, or “high risk of bias”. The evaluation results for each section were summarized and presented in a decision pathway diagram. Reporting quality was evaluated following the CONSORT guidelines [20], covering 6 modules and 37 entries. Depending on the extent to which the literature adhered to each entry, it was rated as “Y” (fully reported), “P” (partially reported), or “N” (not reported). Two investigators assessed the quality of the included studies and cross-validated the evaluation results. Any disagreements were resolved through discussion until a consensus was reached.

### Data analysis

Data were analyzed using STATA 17.0 software to assess the impact of vitamin C supplementation for pregnant smokers during pregnancy on the pulmonary function in the offspring. Weighted Mean Difference (WMD) values and their corresponding 95% confidence intervals (CIs) were computed for continuous variables, including forced expiratory flow at 25% of forced vital capacity (FEF25), forced expiratory flow at 50% of forced vital capacity (FEF50), forced expiratory flow between 25 and 75% (FEF25-75), forced expiratory volume at 0.5 s (FEV0.5), forced vital capacity (FVC), and the ratio of FEV0.5 to FVC (FEV0.5:FVC). Heterogeneity was assessed using the I^2^ statistic and *p*-value. In the case of significant heterogeneity, the random-effects model was employed to analyze the combined WMD; otherwise, a fixed-effects model (FEM) was applied. Sensitivity analysis was conducted by sequentially excluding individual studies. The presence of publication bias was assessed using Egger’s test, with a significance level set at *P*-value < 0.05.

## Result

**Fig. 1:**
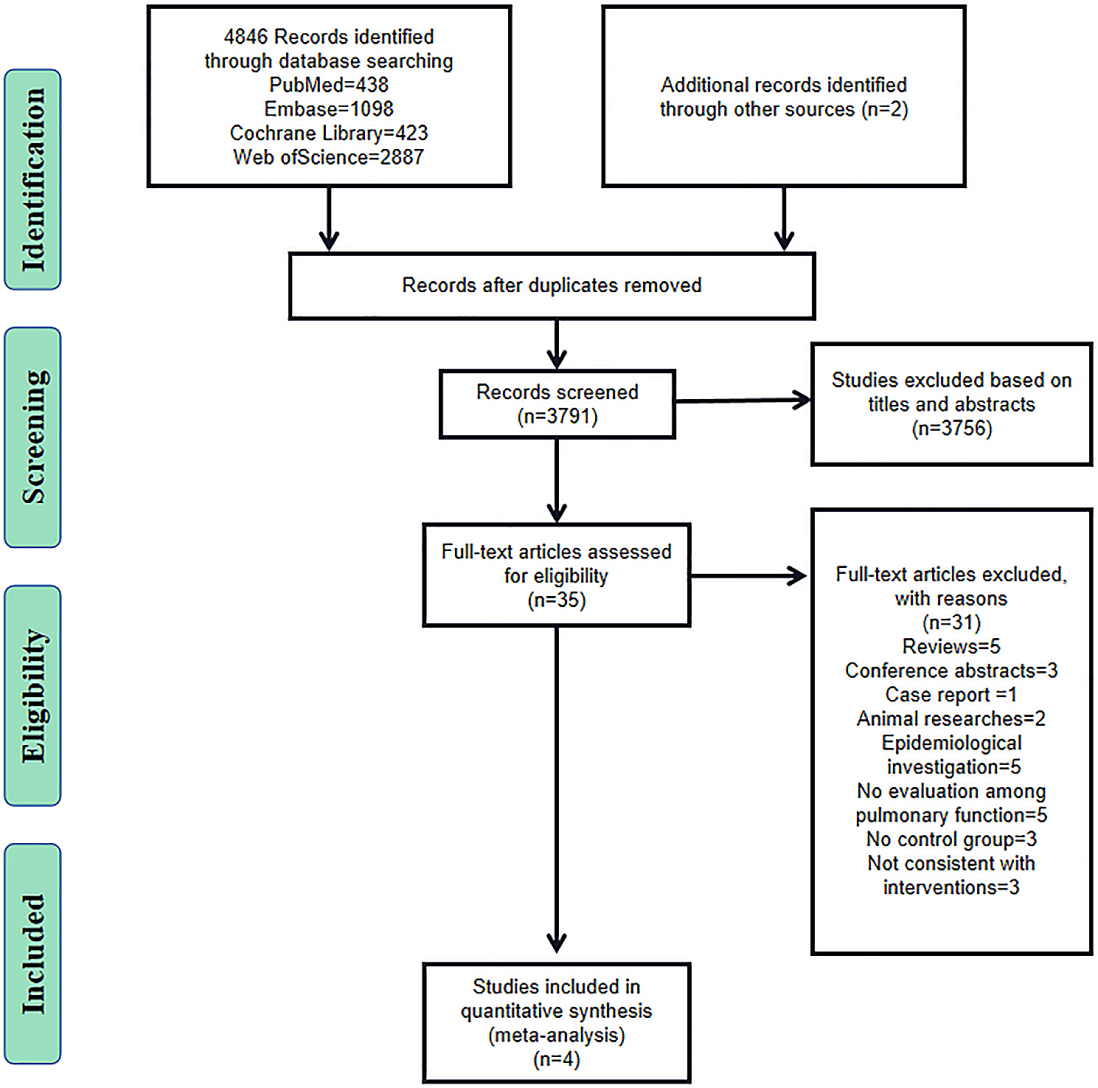
PRISMA (preferred reporting items for systematic reviews and meta-analyses) flow diagram and exclusion criteria

A total of 4,068 studies were retrieved from four databases and references. After removing duplicates, 3,791 studies were further screened based on titles and abstracts. Then the full texts of 35 potentially eligible studies were independently viewed by two investigators. Four studies were ultimately included in this meta-analysis [21–24] (Fig. 1).

The basic characteristics of the included population are shown in Table 1. The number of study subjects in each study ranged from 159 to 222. The reproductive age was between 20 and 32.8 years. The gestational age of the fetus at birth ranged from 36.8 to 41.8 weeks, and the number of cigarettes smoked per day by pregnant women was > 1/d and up to > 7/d.

**Table 1:** Characteristics of included studies (*n* = 4)

Study	Year	Region	Period	Study design	N	Age(Intervention/control)	Smoking status(/d)	Dosage(mg/d)	Infant age(months)	Gestational age, median (weeks)
Cindy T. McEvoy	2014	USA	2007.03-2011.01	RCT	159	26.6 ± 6.2/25.5 ± 5.5	≥ 1	500	0	39.0 ± 2.93/39.1 ± 2.7
Cindy T. McEvoy	2018	USA	2012.12-2015.06	RCT	222	26.6 ± 5.2/26.4 ± 5.9	7.0/7.5	500	3	38.7 ± 1.8/38.6 ± 1.7
Cindy T. McEvoy	2020	USA	2012–2016	RCT	214	26.6 ± 5.3/26.4 ± 5.9	7.0/7.5	500	12	38.7 ± 1.8/38.6 ± 1.7
Cindy T. McEvoy	2022	USA	2018–2021	RCT	192	26.5 ± 5.3/26.7 ± 5.9	7.5/8	500	60	38.7 ± 1.9/38.6 ± 1.7

All included studies were RCTs. Quality assessment was conducted using the ROB 2.0 tool, and the specific results can be found in Fig. S1 and Fig. S2. All studies used blinding procedures in place and followed the established interventions and allocation, without any deviations, resulting in a low risk of bias for each study. Complete outcome data were available for all studies. Despite subject exclusion and dropout in all studies, they had no substantial impact on the results. The assessment of outcome bias revealed that there was 0 (0%) RCT at possible risk and 4 (100%) at low risk. No studies exhibited bias in the selective reporting of results. Overall, all the included articles were deemed to have a low risk of bias. Furthermore, the results of Egger’s test indicated no publication bias (*P* = 0.56) (Fig. S3).

### Pulmonary function

**Fig. 2:**
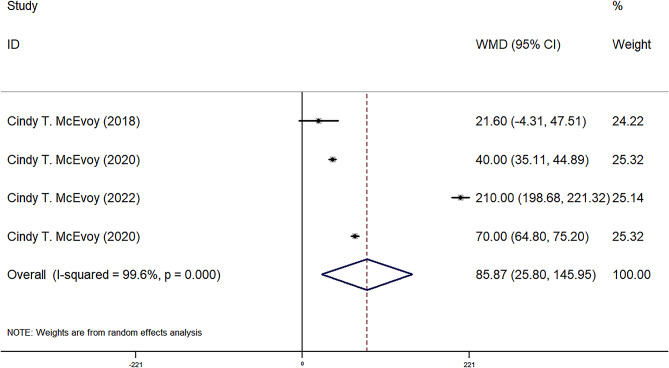
Forest plot for FEF25-75

**Fig. 3:**
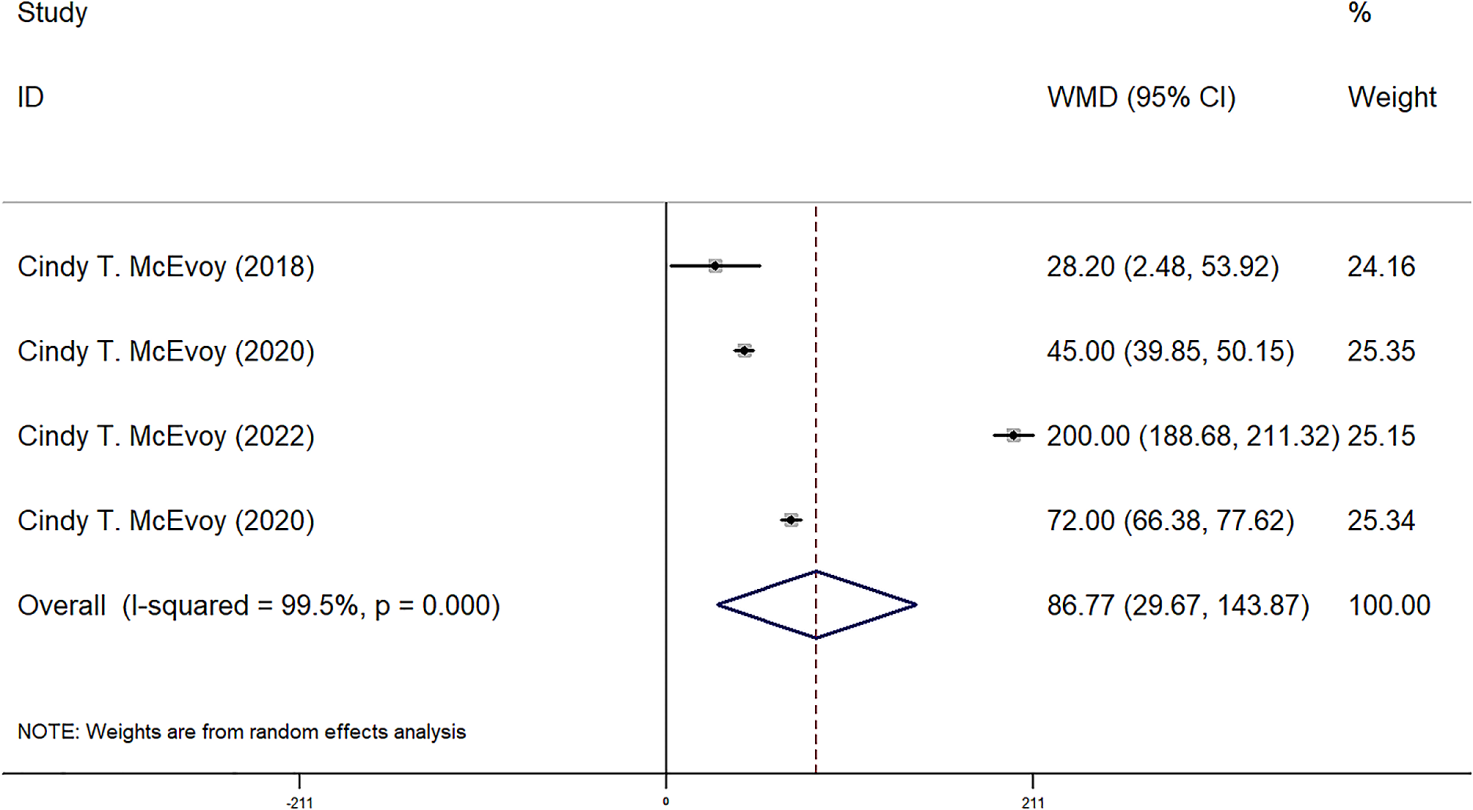
Forest plot for FEF50

**Fig. 4:**
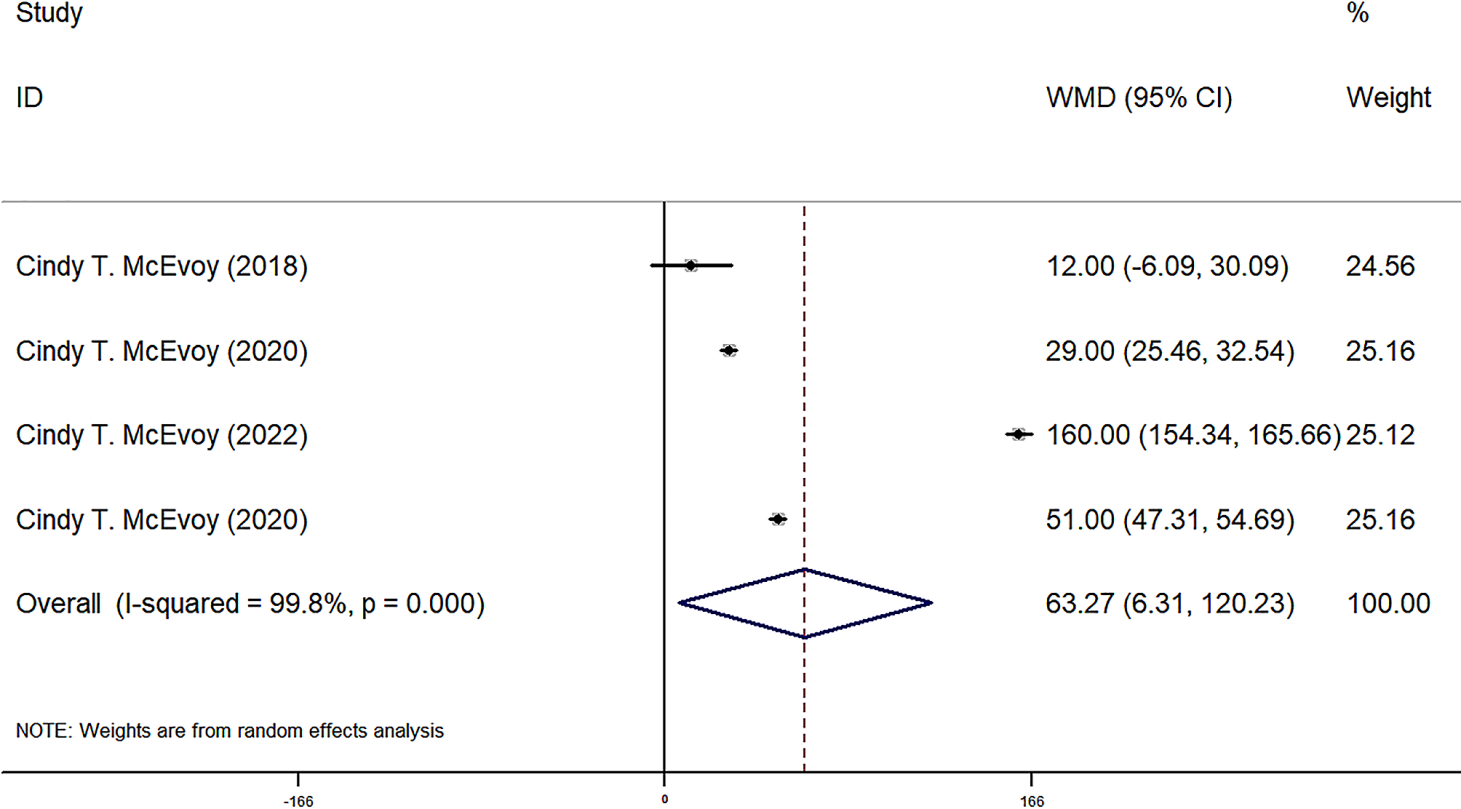
Forest plot for FEF75

**Fig. 5:**
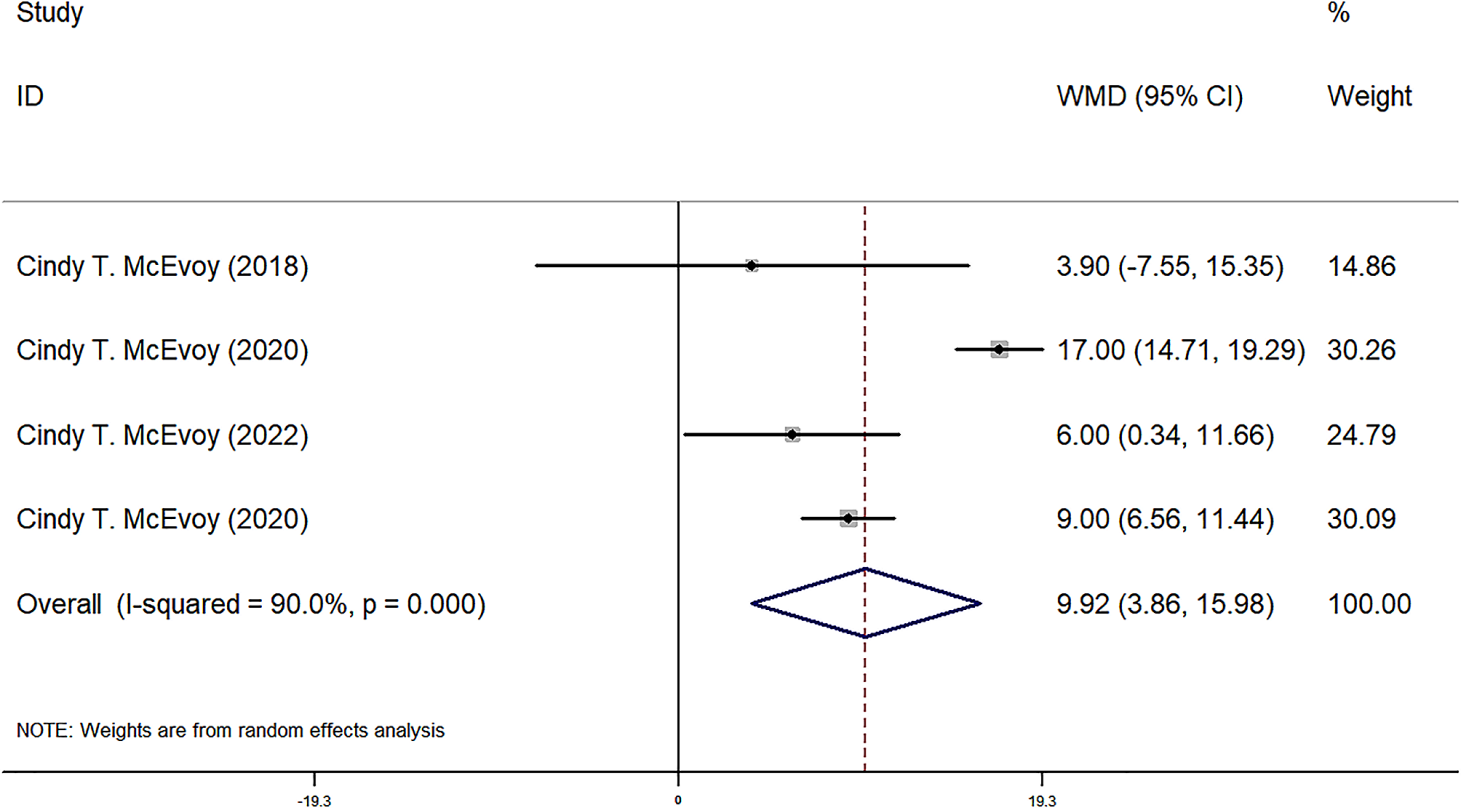
Forest plot for FVC

**Fig. 6:**
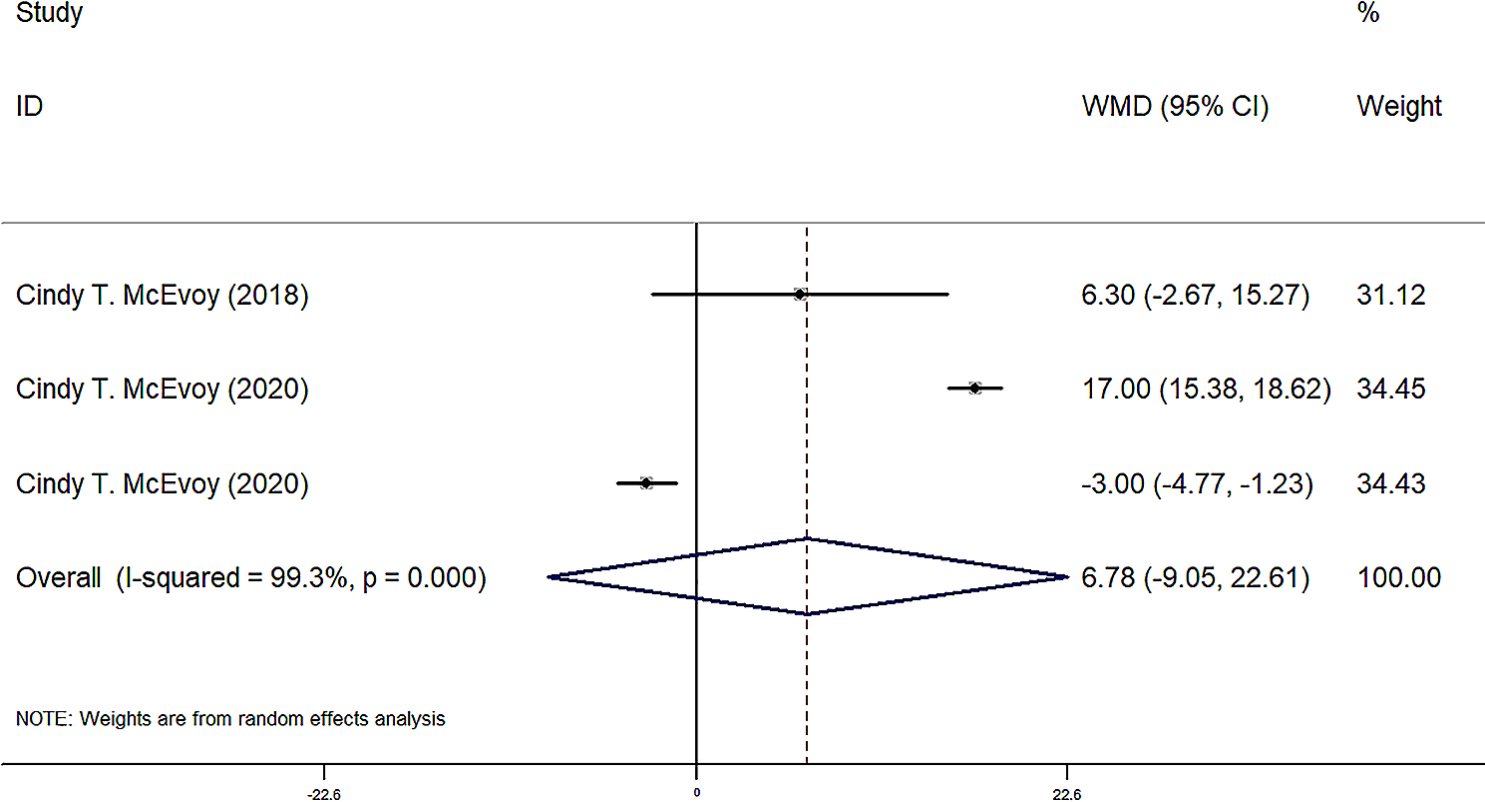
Forest plot for FEV0.5

**Fig. 7:**
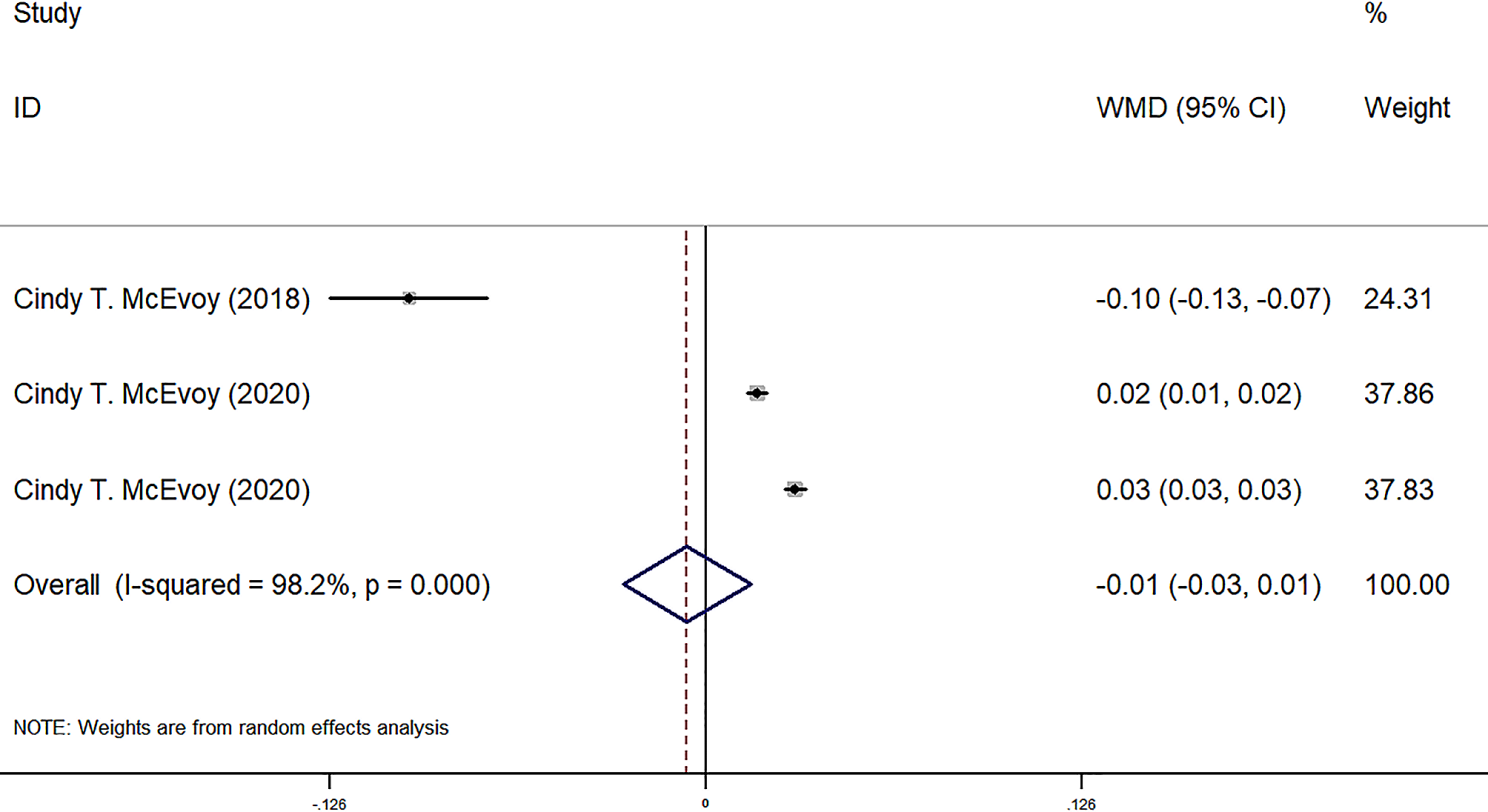
Forest plot for FEV0.5: FVC

FEF25-75, FEF50, FEF75, and FVC were reported in four observational groups across three studies. The results indicated that the offspring of pregnant smokers in the vitamin C group exhibited improved FEF25-75 (WMD = 85.87 ml/sec, 95% CI = 25.80, 145.95, *P* = 0.005) (Fig. 2), FEF50 (WMD = 86.77 ml/sec, 95% CI = 29.67, 143.87, *P* = 0.03) (Fig. 3), FEF75 (WMD = 63.27 ml/sec, 95% CI = 6.31, 120.23, *P* = 0.029) (Fig. 4), and FVC (WMD = 9.92 ml, 95% CI = 3.86, 15.98, *P* = 0.001) (Fig. 5) compared to those in the control group. Additionally, FEV0.5 and the ratio of FEV0.5 to FVC were reported in three observational groups across two studies. However, no statistically significant differences were observed in FEV0.5 (WMD = 6.78 ml, 95% CI=-9.05, 22.61, *P* = 0.401) (Fig. 6) or FEV0.5:FVC (WMD=-0.01, 95% CI=-0.03, 0.01, *P* = 0.552) (Fig. 7) between the vitamin C group and the control group.

## Discussion

This systematic review and meta-analysis illuminated the effect of vitamin C supplementation for pregnant smokers on the pulmonary function of their offspring. The findings revealed that FEF25, FEF50, and FEF25-75 in the vitamin C group were significantly improved compared with those in the control group; the vitamin C group also showed better FVC than the control group; no statistical differences were noted in FEV0.5 and FEV0.5: FVC.

In pulmonary function tests, FEF25, FEF50, and FEF75 represent the instantaneous flow rate at 25%, 50%, and 75% of lung capacity during forceful expiration, respectively. These parameters are widely used to measure airway resistance during respiration. In this study, the results for FEF25, FEF50, and FEF25-75 in the vitamin C group outperformed those in the control group. This suggests that pregnant smokers with vitamin C supplementation show significantly lower airway resistance than controls. This improvement may be attributed to the fact that maternal intake of vitamin C influences placental DNA methylation, which, in turn, affects pulmonary function. Several previous studies have noted that not all cases of maternal smoking significantly increase the incidence of lower respiratory diseases in the offspring [3, 25]. Differences in maternal and fetal genetic susceptibility or epigenetic factors could potentially explain this variation. Some studies [26, 27] have confirmed a close relationship between genotypic differences and pulmonary function in newborns exposed to tobacco. A study by Owens [26] did not show significant differences in pulmonary function in the offspring of patients with GSTM1-active genotypes, regardless of exposure to maternal tobacco. This suggests that the glutathione S-transferase genotype may protect against pulmonary function defects in the context of in-utero tobacco exposure. Shorey-Kendrick [27] measured DNA methylation of placental epigenomes at delivery from 72 pregnant smokers (35 on placebo and 37 on vitamin C) and 24 pregnant women who never smoked. They analyzed the functional enrichment of different methylation loci between groups and indicated that placental DNA methylation was consistently lower in pregnant smokers receiving different placebos. Additionally, eQTM analysis showed that a subset of candidate genes and CpG sites were associated with FEF75 and/or compound wheezing [28].

Furthermore, the offspring born to pregnant smokers in the vitamin C group also exhibited significantly improved FVC compared to the controls. This implies that vitamin C may yield advantageous effects on the overall lung capacity of the offspring born to pregnant smokers. Upon exposure to cigarette smoke actively or passively, individuals are confronted with more than 4,700 complex compounds, many of which are rich in free radicals and other oxidants. These by-products of smoking can trigger intracellular enzymes that generate reactive oxygen species, leading to oxidative stress and lung inflammation. Maity et al. [29] discovered that cigarette smoke could activate the transcriptional activator nuclear factor κB (NF-κB) through a novel transduction pathway of IKKβ-I-κBɛ-c-Rel/p50. Vitamin C, a potent antioxidant, plays a vital role in maintaining the balance of intracellular reactive oxygen levels. Silver et al. [30] found that vitamin C supplementation had an impact on alveolar macrophages and polymorphonuclear cells in mice exposed to cigarette smoke. Furthermore, vitamin C supplementation significantly reduced levels of nuclear P65 in lung tissue and inhibited the activation of NF-κB, ultimately reducing inflammation. In contrast, Das et al. [31] revealed that vitamin C influenced NF-κB through multiple mechanisms. Vitamin C counteracted the degradation of I-κBɛ induced by cigarette smoke extract, which in turn affected c-Rel nuclear translocation and NF-κB activation. This suggests that the mechanisms of vitamin C in pulmonary function in the offspring born to pregnant smokers may vary.

FEV0.5 represents the volume of air forcefully exhaled within 0.5 s after a forceful inhalation relative to the total lung capacity, and FEV0.5:FVC stands for the ratio of FEV0.5 to FVC. These two parameters are commonly used to assess airflow obstruction. This study demonstrated no statistically significant difference in the two indicators, possibly due to little reliable data caused by the challenges of achieving forceful expiration in the offspring and young children. Additionally, vitamin C supplementation for pregnant smokers may not improve airflow obstruction in their offspring. Interestingly, another study [21] conducted by the same research team compared the ratio of functional residual capacity to peak time (TPTEF:TE) at birth and the age of 1 year in the offspring born to pregnant smokers. Their findings revealed that the TPTEF:TE ratio in the offspring of pregnant smokers who received vitamin C supplementation was significantly better than that of the controls. The assessment of pulmonary function indicators in this study required less active cooperation from the children, suggesting that these indicators may have greater clinical practical significance. However, further studies are required to comprehensively understand the effect of vitamin C supplementation on the pulmonary function of the offspring born to pregnant smokers, as both FEV0.5 and FEV0.5:FVC are crucial for evaluating pulmonary function.

The high variability in the outcome indicators of this study may be attributed to several factors. First, few high-quality RCTs have examined the effects of vitamin C supplementation for pregnant smokers on the pulmonary function of their offspring. The small sample size in these studies could significantly impact the findings. Second, the age span of the study population is large, resulting in notable differences in pulmonary function indicators among offspring and young children in different age groups. This diversity in age groups may contribute to the heterogeneity. Third, owing to the young age of the study population, it was difficult to achieve full cooperation during the pulmonary function tests. Consequently, there was substantial variability in the results of these tests, which may also account for the observed heterogeneity [32].

This meta-analysis also has several limitations. First, there are few studies on vitamin C supplementation for pregnant smokers. Consequently, this meta-analysis had to rely on findings from various articles published by the same research team, which may have biased the results. Second, as pulmonary function data were collected from the same group of individuals at different ages, the overall sample size was small, and the age range was wide. This resulted in increased variation in test indicators across different age groups, leading to inherent high heterogeneity. However, it remains crucial to explore whether vitamin C supplementation for pregnant women who smoke can enhance the pulmonary function of their offspring. This is particularly important in light of the continued high prevalence of pregnant smokers worldwide [1] and the detrimental effects of smoking on the well-being of both pregnant women and their offspring. Therefore, it is imperative to further illuminate the role of vitamin C supplementation in improving the pulmonary function of the offspring born to pregnant smokers, so as to provide a novel approach to mitigate the impact of maternal smoking on the lung health of offspring.

## Conclusion

Vitamin C supplementation for pregnant smokers may be effective in improving vital capacity and respiratory airway resistance in the offspring, whereas improvement in FEV1 is debatable. The present meta-analysis provides new insights into whether pregnant smokers should be supplemented with vitamin C to improve the pulmonary function in their offspring.

### Electronic supplementary material

Below is the link to the electronic supplementary material.

Supplementary Material 1

Supplementary Material 2

## Data Availability

All data generated or analyzed during this study are included in this published article and its supplementary information files.

## Abbreviations

OR: Odd ratio
WMD: Weighted Mean Difference
CI: Confidence interval
BMI: Body Mass Index
FEF25-75: Forced Expiratory Flow between 25 and 75%
FEF50: Forced Expiratory Flow at 50%
FEF75: Forced Expiratory Flow at 75%
FVC: Forced Vital Capacity
FEV0.5: Forced Expiratory Volume at 0.5 s
