# Supplementary material for: A meta-analysis of the effects of vitamin C supplementation for pregnant smokers on the pulmonary function of their offspring
Source: BMC Pregnancy Childbirth. 2024 Mar 7;24:184. doi: 10.1186/s12884-024-06377-3 (PMC10921735; doi:10.1186/s12884-024-06377-3)
Supplement: Supplementary file 1 — Supplementary Material 1 [file 12884_2024_6377_MOESM1_ESM.docx]

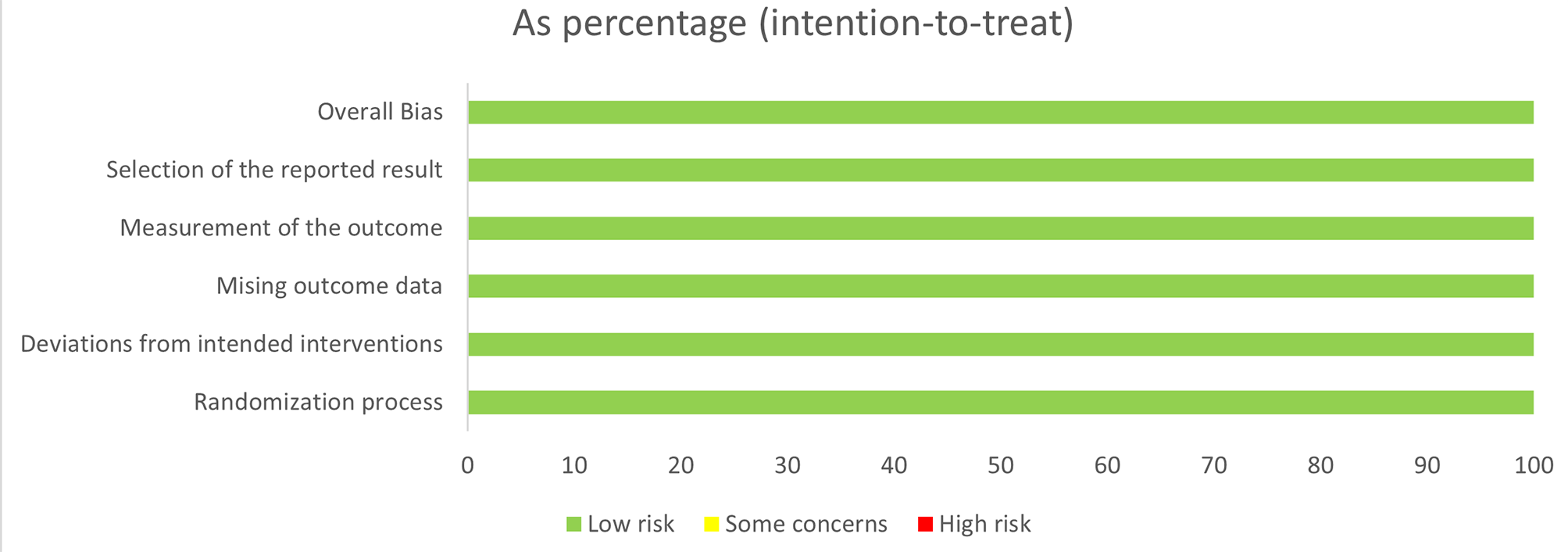


**Fig. S1** Assignment to intervention (the 'intention-to-treat' effect)


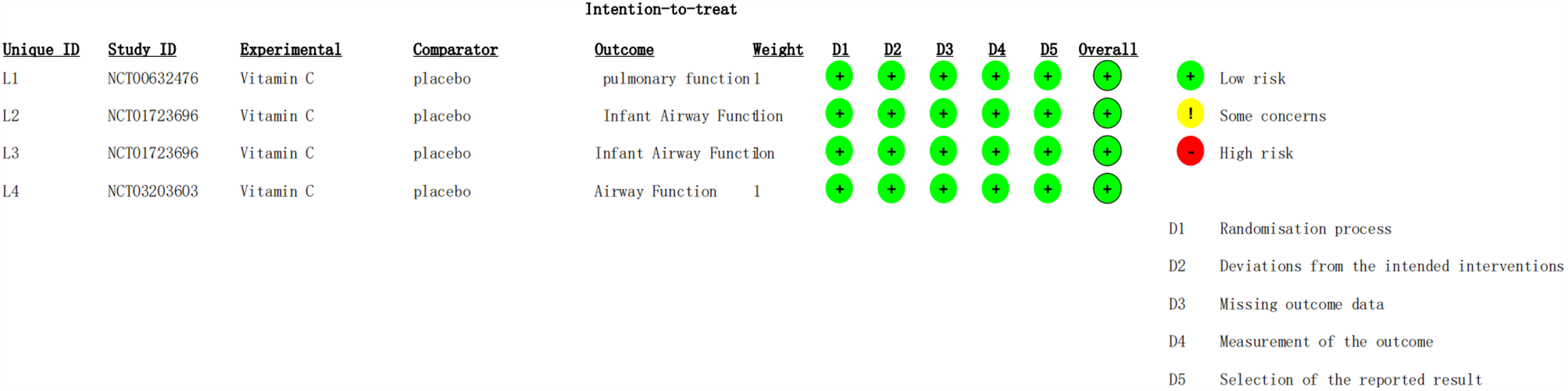


**Fig. S2** Results of literature quality evaluation


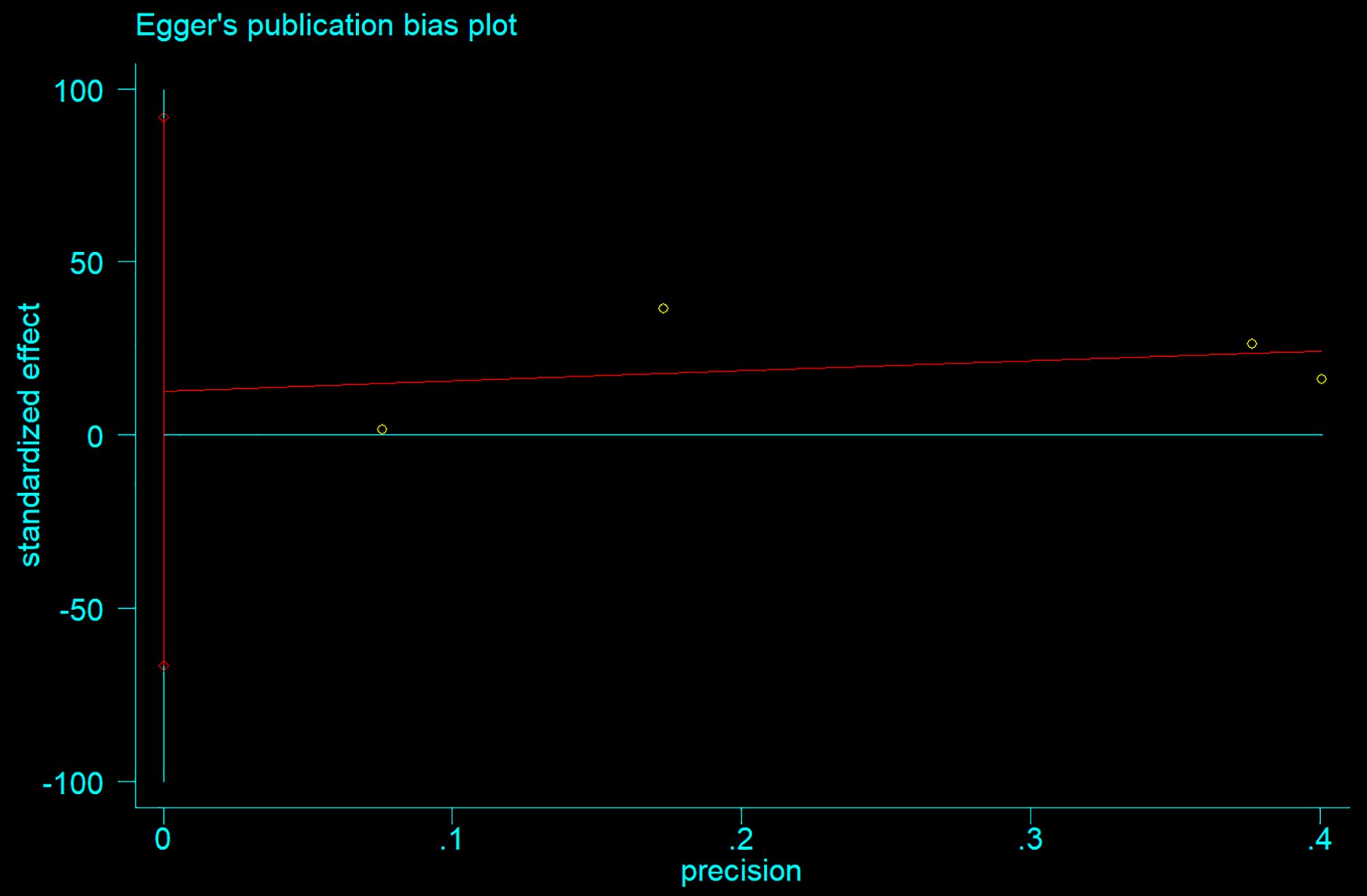


**Fig. S3** The result of the Egger’s test.
