# Supplementary material for: A meta-analysis of the effects of vitamin C supplementation for pregnant smokers on the pulmonary function of their offspring
Source: BMC Pregnancy Childbirth. 2024 Mar 7;24:184. doi: 10.1186/s12884-024-06377-3 (PMC10921735; doi:10.1186/s12884-024-06377-3)
Supplement: Supplementary file 2 — Supplementary Material 2 [file 12884_2024_6377_MOESM2_ESM.docx]

**Supplementary material 1** Document retrieval strategy

**Cochrane**

Search Name:

Date Run: 08/04/2023 16:51:37

Comment:

ID Search Hits

#1 'Acid, Ascorbic' OR 'Acid, L-Ascorbic' OR 'Ascorbate, Ferrous' OR 'Ascorbate, Magnesium' OR 'Ascorbate, Sodium' OR 'Ascorbic Acid' OR 'Ascorbic Acid, Monosodium Salt' OR 'di-L-Ascorbate, Magnesium' OR 'Ferrous Ascorbate' OR 'Hybrin' OR 'L Ascorbic Acid' OR 'L-Ascorbic Acid' OR 'Magnesium Ascorbate' OR 'Magnesium Ascorbicum' OR 'Magnesium di L Ascorbate' OR 'Magnesium di-L-Ascorbate' OR 'Magnorbin' OR 'Sodium Ascorbate' OR 'Vitamin C' 14710

#2 MeSH descriptor: [Ascorbic Acid] explode all trees 2566

#3 ('pregnant woman' OR 'pregnant women'):ti,ab,kw 22159

#4 MeSH descriptor: [Pregnant Women] explode all trees 725

#5 (#1 OR #2) AND (#3 OR #4) 506

**Embase queries**

| No. | Query | Results | Date |
| --- | --- | --- | --- |
| #5 | (#1 OR #2) AND (#3 OR #4) | 1098 | 8-Apr-23 |
| #4 | 'pregnant woman'/exp | 107528 | 8-Apr-23 |
| #3 | 'pregnant woman':ti,ab,kw OR 'pregnant women':ti,ab,kw | 166668 | 8-Apr-23 |
| #2 | 'ascorbic acid'/exp | 115605 | 8-Apr-23 |
| #1 | acid, ascorbic':ti,ab,kw OR 'acid, l ascorbic':ti,ab,kw OR 'acidum ascorbicum':ti,ab,kw OR 'acidylina':ti,ab,kw OR 'adenex':ti,ab,kw OR 'afj c':ti,ab,kw OR 'agrumina':ti,ab,kw OR 'allercorb':ti,ab,kw OR 'allescorb':ti,ab,kw OR 'antiscorbutic vitamin':ti,ab,kw OR 'arcavit c':ti,ab,kw OR 'arcavite c':ti,ab,kw OR 'arkovital c':ti,ab,kw OR 'ascelat':ti,ab,kw OR 'ascofar':ti,ab,kw OR 'ascomed':ti,ab,kw OR 'asconvita':ti,ab,kw OR 'ascor':ti,ab,kw OR 'ascor l 500':ti,ab,kw OR 'ascorbate':ti,ab,kw OR 'ascorbate sodium':ti,ab,kw OR 'ascorbic acid':ti,ab,kw OR 'ascorbic acid potassium salt':ti,ab,kw OR 'ascorbic acid, monosodium salt':ti,ab,kw OR 'ascorbicap':ti,ab,kw OR 'ascorbicin':ti,ab,kw OR 'ascorbico':ti,ab,kw OR 'ascorbin':ti,ab,kw OR 'ascorbina':ti,ab,kw OR 'ascorbinic acid':ti,ab,kw OR 'ascorbit':ti,ab,kw OR 'ascorbite':ti,ab,kw OR 'ascorbitol':ti,ab,kw OR 'ascorbivit':ti,ab,kw OR 'ascorbivite':ti,ab,kw OR 'ascorbone':ti,ab,kw OR 'ascorbutina':ti,ab,kw OR 'ascorbyl':ti,ab,kw OR 'ascorbyn':ti,ab,kw OR 'ascorcee':ti,ab,kw OR 'ascorgil':ti,ab,kw OR 'ascorin':ti,ab,kw OR 'ascormin':ti,ab,kw OR 'ascorteal':ti,ab,kw OR 'ascorval':ti,ab,kw OR 'ascorvel':ti,ab,kw OR 'ascorvit':ti,ab,kw OR 'ascorvite':ti,ab,kw OR 'ascorvitina':ti,ab,kw OR 'askorbin':ti,ab,kw OR 'austrovit c':ti,ab,kw OR 'austrovite c':ti,ab,kw OR 'bentavit c':ti,ab,kw OR 'bentavite c':ti,ab,kw OR 'c crivit':ti,ab,kw OR 'c ine':ti,ab,kw OR 'c level':ti,ab,kw OR 'c lisa':ti,ab,kw OR 'c long':ti,ab,kw OR 'c monovit':ti,ab,kw OR 'c monovite':ti,ab,kw OR 'c prana':ti,ab,kw OR 'c rivitin':ti,ab,kw OR 'c rivitine':ti,ab,kw OR 'c sol':ti,ab,kw OR 'c tamin':ti,ab,kw OR 'c tamine':ti,ab,kw OR 'c tard':ti,ab,kw OR 'c tonic':ti,ab,kw OR 'c tron':ti,ab,kw OR 'c vescent':ti,ab,kw OR 'c vicotrat':ti,ab,kw OR 'c vicotrate':ti,ab,kw OR 'c vimin':ti,ab,kw OR 'c vimine':ti,ab,kw OR 'c vit':ti,ab,kw OR 'c vita':ti,ab,kw OR 'c vital':ti,ab,kw OR 'c vitam':ti,ab,kw OR 'c vite':ti,ab,kw OR 'c will':ti,ab,kw OR 'cantan':ti,ab,kw OR 'cantaxin':ti,ab,kw OR 'catavin c':ti,ab,kw OR 'ce arom':ti,ab,kw OR 'ce limo':ti,ab,kw OR 'ce major':ti,ab,kw OR 'ce quin':ti,ab,kw OR 'ce quine':ti,ab,kw OR 'ce vi sol':ti,ab,kw OR 'ce vita':ti,ab,kw OR 'ce vitan':ti,ab,kw OR 'cebetate':ti,ab,kw OR 'cebicure':ti,ab,kw OR 'cebiolon':ti,ab,kw OR 'cebion':ti,ab,kw OR 'cebione':ti,ab,kw OR 'cecap':ti,ab,kw OR 'cecon':ti,ab,kw OR 'cecon drops':ti,ab,kw OR 'cecon solution':ti,ab,kw OR 'cecone':ti,ab,kw OR 'cecorbin':ti,ab,kw OR 'cecorbine':ti,ab,kw OR 'cecorbyl':ti,ab,kw OR 'cecorbyle':ti,ab,kw OR 'cecrisina':ti,ab,kw OR 'cedon':ti,ab,kw OR 'cedone':ti,ab,kw OR 'cedoxon':ti,ab,kw OR 'cedoxone':ti,ab,kw OR 'cee 500':ti,ab,kw OR 'ceevifil':ti,ab,kw OR 'cegiolan':ti,ab,kw OR 'celaskon':ti,ab,kw OR 'celaskone':ti,ab,kw OR 'celin':ti,ab,kw OR 'celine':ti,ab,kw OR 'cenetone':ti,ab,kw OR 'cenol':ti,ab,kw OR 'cenolate':ti,ab,kw OR 'cequinyl':ti,ab,kw OR 'cereon':ti,ab,kw OR 'cergona':ti,ab,kw OR 'cescorbat':ti,ab,kw OR 'cetamican':ti,ab,kw OR 'cetamid':ti,ab,kw OR 'cetamin knoll australia':ti,ab,kw OR 'cetamine':ti,ab,kw OR 'cetamine knoll australia':ti,ab,kw OR 'cetebe':ti,ab,kw OR 'ceterapion':ti,ab,kw OR 'ceterapione':ti,ab,kw OR 'cetrinets':ti,ab,kw OR 'cevalin':ti,ab,kw OR 'cevaline':ti,ab,kw OR 'cevatine':ti,ab,kw OR 'cevex':ti,ab,kw OR 'cevibram':ti,ab,kw OR 'cevigal':ti,ab,kw OR 'cevigen':ti,ab,kw OR 'cevigol':ti,ab,kw OR 'cevikap':ti,ab,kw OR 'cevilat':ti,ab,kw OR 'cevimin':ti,ab,kw OR 'cevimine':ti,ab,kw OR 'cevisol':ti,ab,kw OR 'cevit':ti,ab,kw OR 'cevita':ti,ab,kw OR 'cevitamic acid':ti,ab,kw OR 'cevitamin':ti,ab,kw OR 'cevitaminic acid':ti,ab,kw OR 'cevitaminum kolin':ti,ab,kw OR 'cevitan':ti,ab,kw OR 'cevite':ti,ab,kw OR 'cevitex':ti,ab,kw OR 'cevitil':ti,ab,kw OR 'cevitol':ti,ab,kw OR 'cewin':ti,ab,kw OR 'chewcee':ti,ab,kw OR 'chivibit c':ti,ab,kw OR 'ci drol':ti,ab,kw OR 'ciamin':ti,ab,kw OR 'ciamina ormo':ti,ab,kw OR 'ciergin':ti,ab,kw OR 'cifilina':ti,ab,kw OR 'cipca':ti,ab,kw OR 'cisir':ti,ab,kw OR 'citamino':ti,ab,kw OR 'cith':ti,ab,kw OR 'citoascorbina':ti,ab,kw OR 'citoxyl':ti,ab,kw OR 'citran':ti,ab,kw OR 'citravite':ti,ab,kw OR 'citritabs':ti,ab,kw OR 'citrovitamina':ti,ab,kw OR 'civigor':ti,ab,kw OR 'civitin':ti,ab,kw OR 'civitine':ti,ab,kw OR 'co biagini':ti,ab,kw OR 'concemin':ti,ab,kw OR 'cortalex':ti,ab,kw OR 'd ascorbic acid':ti,ab,kw OR 'd xyloascorbic acid':ti,ab,kw OR 'dagrascorbin':ti,ab,kw OR 'dagravit c':ti,ab,kw OR 'dancimin c':ti,ab,kw OR 'davitamon c':ti,ab,kw OR 'dayvital':ti,ab,kw OR 'delo c':ti,ab,kw OR 'difvitamin c':ti,ab,kw OR 'dropice':ti,ab,kw OR 'dumovit c':ti,ab,kw OR 'dumovite c':ti,ab,kw OR 'e ascorbic acid':ti,ab,kw OR 'e xyloascorbic acid':ti,ab,kw OR 'erftamin c':ti,ab,kw OR 'erftamine c':ti,ab,kw OR 'esuron':ti,ab,kw OR 'esurvit':ti,ab,kw OR 'esurvite':ti,ab,kw OR 'ferrous ascorbate':ti,ab,kw OR 'flavettes':ti,ab,kw OR 'godabion c':ti,ab,kw OR 'gregovite c':ti,ab,kw OR 'hicee':ti,ab,kw OR 'hybrin':ti,ab,kw OR 'ido c':ti,ab,kw OR 'ikacee':ti,ab,kw OR 'inovitan c':ti,ab,kw OR 'irocevit':ti,ab,kw OR 'irocevite':ti,ab,kw OR 'jarexin':ti,ab,kw OR 'jarexine':ti,ab,kw OR 'l 3 keto hexuronic acid lactone':ti,ab,kw OR 'l ascorbic acid':ti,ab,kw OR 'l xylo ascorbic acid':ti,ab,kw OR 'l xyloascorbic acid':ti,ab,kw OR 'lacivit':ti,ab,kw OR 'lacivite':ti,ab,kw OR 'laroscorbine':ti,ab,kw OR 'leder c':ti,ab,kw OR 'lemascorb':ti,ab,kw OR 'levo ascorbate':ti,ab,kw OR 'levo ascorbic acid':ti,ab,kw OR 'limcee':ti,ab,kw OR 'limo ce':ti,ab,kw OR 'liqui cee':ti,ab,kw OR 'magnesium ascorbate':ti,ab,kw OR 'magnesium ascorbicum':ti,ab,kw OR 'magnesium di l ascorbate':ti,ab,kw OR 'magnorbin':ti,ab,kw OR 'mega c/a plus':ti,ab,kw OR 'myascorbin':ti,ab,kw OR 'natrascorb':ti,ab,kw OR 'novo ascorbic':ti,ab,kw OR 'nybadol':ti,ab,kw OR 'paa 500':ti,ab,kw OR 'parkovit c':ti,ab,kw OR 'pascorbin':ti,ab,kw OR 'pharmascorbine':ti,ab,kw OR 'pharmatovit c':ti,ab,kw OR 'pharmatovite c':ti,ab,kw OR 'planavit c':ti,ab,kw OR 'planavite c':ti,ab,kw OR 'plivit c':ti,ab,kw OR 'plivite c':ti,ab,kw OR 'potassium ascorbate':ti,ab,kw OR 'pro c':ti,ab,kw OR 'proscorbin':ti,ab,kw OR 'proscorbine':ti,ab,kw OR 'redoxon':ti,ab,kw OR 'redoxon c':ti,ab,kw OR 'redoxon forte':ti,ab,kw OR 'ribena':ti,ab,kw OR 'scorbacid':ti,ab,kw OR 'scorbacide':ti,ab,kw OR 'scorbettes':ti,ab,kw OR 'scorbex':ti,ab,kw OR 'scorbin c':ti,ab,kw OR 'scorbitol':ti,ab,kw OR 'scorbumine':ti,ab,kw OR 'scottavit c':ti,ab,kw OR 'scottavite c':ti,ab,kw OR 'secorbate':ti,ab,kw OR 'sevalin':ti,ab,kw OR 'sigmavit c':ti,ab,kw OR 'sigmavite c':ti,ab,kw OR 'sodascorbate':ti,ab,kw OR 'sodium ascorbate':ti,ab,kw OR 'sodium l ascorbate':ti,ab,kw OR 'sweetcee':ti,ab,kw OR 'synum c':ti,ab,kw OR 'take c':ti,ab,kw OR 'tanvimil c':ti,ab,kw OR 'testascorbic':ti,ab,kw OR 'ucemine c':ti,ab,kw OR 'upsa c':ti,ab,kw OR 'upsavit':ti,ab,kw OR 'vagi c':ti,ab,kw OR 'vi c 500':ti,ab,kw OR 'vi ci sin':ti,ab,kw OR 'vi dom c':ti,ab,kw OR 'vicef':ti,ab,kw OR 'vicelat':ti,ab,kw OR 'vicetrin':ti,ab,kw OR 'vici monico':ti,ab,kw OR 'viciman':ti,ab,kw OR 'vicin':ti,ab,kw OR 'vicitina':ti,ab,kw OR 'vicon':ti,ab,kw OR 'viforcit':ti,ab,kw OR 'viforcite':ti,ab,kw OR 'viscorin':ti,ab,kw OR 'viscorine':ti,ab,kw OR 'vita cedol orange':ti,ab,kw OR 'vita gem c':ti,ab,kw OR 'vitac':ti,ab,kw OR 'vitace':ti,ab,kw OR 'vitacee':ti,ab,kw OR 'vitaci':ti,ab,kw OR 'vitacimin':ti,ab,kw OR 'vitacimine':ti,ab,kw OR 'vitacin':ti,ab,kw OR 'vitacine':ti,ab,kw OR 'vitamin c':ti,ab,kw OR 'vitamin c 1':ti,ab,kw OR 'vitaplex c':ti,ab,kw OR 'vitapric':ti,ab,kw OR 'vitapur c':ti,ab,kw OR 'vitasan c':ti,ab,kw OR 'vitascorbin':ti,ab,kw OR 'vitascorbine':ti,ab,kw OR 'vitascorbol':ti,ab,kw OR 'vitascorbol 500':ti,ab,kw OR 'vitelix c':ti,ab,kw OR 'vitocee':ti,ab,kw OR 'vorange':ti,ab,kw OR 'wandervit c':ti,ab,kw OR 'wandervite c':ti,ab,kw OR 'witamina c':ti,ab,kw OR 'xitix':ti,ab,kw OR 'xon ce':ti,ab,kw OR 'xyloascorbic acid':ti,ab,kw | 98184 | 8-Apr-23 |

**PubMedSearchHistory：**

| Search number | Query | Sort By | Filters | Search Details | Results | Time |
| --- | --- | --- | --- | --- | --- | --- |
| 5 | (#1 OR #2) AND (#3 OR #4) |  |  | ("acid ascorbic"[Title/Abstract] OR "acid l ascorbic"[Title/Abstract] OR "acidum ascorbicum"[Title/Abstract] OR "adenex"[Title/Abstract] OR "allercorb"[Title/Abstract] OR "antiscorbutic vitamin"[Title/Abstract] OR "ascor"[Title/Abstract] OR "ascorbate"[Title/Abstract] OR "ascorbate sodium"[Title/Abstract] OR "Ascorbic Acid"[Title/Abstract] OR "ascorbicap"[Title/Abstract] OR "ascorbico"[Title/Abstract] OR "ascorbin"[Title/Abstract] OR "ascorbinic acid"[Title/Abstract] OR "ascorbit"[Title/Abstract] OR "ascorbone"[Title/Abstract] OR "ascorbutina"[Title/Abstract] OR "ascorbyl"[Title/Abstract] OR "ascorin"[Title/Abstract] OR "ascorteal"[Title/Abstract] OR "ascorvit"[Title/Abstract] OR "c level"[Title/Abstract] OR "c long"[Title/Abstract] OR "c sol"[Title/Abstract] OR "c tamin"[Title/Abstract] OR "c tonic"[Title/Abstract] OR "c vimin"[Title/Abstract] OR "c vit"[Title/Abstract] OR "c vita"[Title/Abstract] OR "c vital"[Title/Abstract] OR "c will"[Title/Abstract] OR "cantan"[Title/Abstract] OR "cantaxin"[Title/Abstract] OR "catavin c"[Title/Abstract] OR "ce vi sol"[Title/Abstract] OR "cebicure"[Title/Abstract] OR "cebion"[Title/Abstract] OR "cebione"[Title/Abstract] OR "cecap"[Title/Abstract] OR "cecon"[Title/Abstract] OR "cee 500"[Title/Abstract] OR "cegiolan"[Title/Abstract] OR "celaskon"[Title/Abstract] OR "celin"[Title/Abstract] OR "celine"[Title/Abstract] OR "cenetone"[Title/Abstract] OR "cenolate"[Title/Abstract] OR "cereon"[Title/Abstract] OR "cergona"[Title/Abstract] OR "cescorbat"[Title/Abstract] OR "cetamid"[Title/Abstract] OR "cetamine"[Title/Abstract] OR "cetebe"[Title/Abstract] OR "cevalin"[Title/Abstract] OR "cevaline"[Title/Abstract] OR "cevatine"[Title/Abstract] OR "cevex"[Title/Abstract] OR "cevimin"[Title/Abstract] OR "cevisol"[Title/Abstract] OR "cevit"[Title/Abstract] OR "cevitamic acid"[Title/Abstract] OR "cevitamin"[Title/Abstract] OR "cevitan"[Title/Abstract] OR "cevitex"[Title/Abstract] OR "cewin"[Title/Abstract] OR "ciamin"[Title/Abstract] OR "cipca"[Title/Abstract] OR "cisir"[Title/Abstract] OR "citamino"[Title/Abstract] OR "cith"[Title/Abstract] OR "citran"[Title/Abstract] OR "concemin"[Title/Abstract] OR "cortalex"[Title/Abstract] OR "d ascorbic acid"[Title/Abstract] OR "davitamon c"[Title/Abstract] OR "Ferrous Ascorbate"[Title/Abstract] OR "hicee"[Title/Abstract] OR "Hybrin"[Title/Abstract] OR "ido c"[Title/Abstract] OR "L Ascorbic Acid"[Title/Abstract] OR "l xyloascorbic acid"[Title/Abstract] OR "laroscorbine"[Title/Abstract] OR "lemascorb"[Title/Abstract] OR "levo ascorbic acid"[Title/Abstract] OR "liqui cee"[Title/Abstract] OR "Magnesium Ascorbate"[Title/Abstract] OR "natrascorb"[Title/Abstract] OR "pascorbin"[Title/Abstract] OR "planavit c"[Title/Abstract] OR "potassium ascorbate"[Title/Abstract] OR "pro c"[Title/Abstract] OR "proscorbin"[Title/Abstract] OR "redoxon"[Title/Abstract] OR "ribena"[Title/Abstract] OR "scorbacid"[Title/Abstract] OR "secorbate"[Title/Abstract] OR "Sodium Ascorbate"[Title/Abstract] OR "sodium l ascorbate"[Title/Abstract] OR "testascorbic"[Title/Abstract] OR "vagi c"[Title/Abstract] OR "vicelat"[Title/Abstract] OR "vicin"[Title/Abstract] OR "vicon"[Title/Abstract] OR "viforcit"[Title/Abstract] OR "viscorin"[Title/Abstract] OR "vitac"[Title/Abstract] OR "vitace"[Title/Abstract] OR "vitacee"[Title/Abstract] OR "vitacimin"[Title/Abstract] OR "vitacin"[Title/Abstract] OR "Vitamin C"[Title/Abstract] OR "vitamin c 1"[Title/Abstract] OR "vitascorbol"[Title/Abstract] OR "witamina c"[Title/Abstract] OR "xitix"[Title/Abstract] OR "xyloascorbic acid"[Title/Abstract] OR "Ascorbic Acid"[MeSH Terms]) AND ("pregnant woman"[Title/Abstract] OR "pregnant women"[Title/Abstract] OR "pregnant women"[MeSH Terms]) | 438 | 9:45:35 |
| 4 | Pregnant Women[MeSH Terms] |  |  | "pregnant women"[MeSH Terms] | 13,951 | 9:42:23 |
| 3 | "pregnant woman"[Title/Abstract] OR "pregnant women"[Title/Abstract] |  |  | "pregnant woman"[Title/Abstract] OR "pregnant women"[Title/Abstract] | 122,524 | 9:42:02 |
| 2 | Acid, Ascorbic[MeSH Terms] |  |  | "ascorbic acid"[MeSH Terms] | 45,204 | 9:40:26 |
| 1 | "Acid, Ascorbic"[Title/Abstract] OR "Acid, L Ascorbic"[Title/Abstract] OR "acidum ascorbicum"[Title/Abstract] OR "acidylina"[Title/Abstract] OR "adenex"[Title/Abstract] OR "afj c"[Title/Abstract] OR "agrumina"[Title/Abstract] OR "allercorb"[Title/Abstract] OR "allescorb"[Title/Abstract] OR "antiscorbutic vitamin"[Title/Abstract] OR "arcavit c"[Title/Abstract] OR "arcavite c"[Title/Abstract] OR "arkovital c"[Title/Abstract] OR "ascelat"[Title/Abstract] OR "ascofar"[Title/Abstract] OR "ascomed"[Title/Abstract] OR "asconvita"[Title/Abstract] OR "ascor"[Title/Abstract] OR "ascor l 500"[Title/Abstract] OR "ascorbate"[Title/Abstract] OR "ascorbate sodium"[Title/Abstract] OR "Ascorbic Acid"[Title/Abstract] OR "ascorbic acid potassium salt"[Title/Abstract] OR "Ascorbic Acid, Monosodium Salt"[Title/Abstract] OR "ascorbicap"[Title/Abstract] OR "ascorbicin"[Title/Abstract] OR "ascorbico"[Title/Abstract] OR "ascorbin"[Title/Abstract] OR "ascorbina"[Title/Abstract] OR "ascorbinic acid"[Title/Abstract] OR "ascorbit"[Title/Abstract] OR "ascorbite"[Title/Abstract] OR "ascorbitol"[Title/Abstract] OR "ascorbivit"[Title/Abstract] OR "ascorbivite"[Title/Abstract] OR "ascorbone"[Title/Abstract] OR "ascorbutina"[Title/Abstract] OR "ascorbyl"[Title/Abstract] OR "ascorbyn"[Title/Abstract] OR "ascorcee"[Title/Abstract] OR "ascorgil"[Title/Abstract] OR "ascorin"[Title/Abstract] OR "ascormin"[Title/Abstract] OR "ascorteal"[Title/Abstract] OR "ascorval"[Title/Abstract] OR "ascorvel"[Title/Abstract] OR "ascorvit"[Title/Abstract] OR "ascorvite"[Title/Abstract] OR "ascorvitina"[Title/Abstract] OR "askorbin"[Title/Abstract] OR "austrovit c"[Title/Abstract] OR "austrovite c"[Title/Abstract] OR "bentavit c"[Title/Abstract] OR "bentavite c"[Title/Abstract] OR "c crivit"[Title/Abstract] OR "c ine"[Title/Abstract] OR "c level"[Title/Abstract] OR "c lisa"[Title/Abstract] OR "c long"[Title/Abstract] OR "c monovit"[Title/Abstract] OR "c monovite"[Title/Abstract] OR "c prana"[Title/Abstract] OR "c rivitin"[Title/Abstract] OR "c rivitine"[Title/Abstract] OR "c sol"[Title/Abstract] OR "c tamin"[Title/Abstract] OR "c tamine"[Title/Abstract] OR "c tard"[Title/Abstract] OR "c tonic"[Title/Abstract] OR "c tron"[Title/Abstract] OR "c vescent"[Title/Abstract] OR "c vicotrat"[Title/Abstract] OR "c vicotrate"[Title/Abstract] OR "c vimin"[Title/Abstract] OR "c vimine"[Title/Abstract] OR "c vit"[Title/Abstract] OR "c vita"[Title/Abstract] OR "c vital"[Title/Abstract] OR "c vitam"[Title/Abstract] OR "c vite"[Title/Abstract] OR "c will"[Title/Abstract] OR "cantan"[Title/Abstract] OR "cantaxin"[Title/Abstract] OR "catavin c"[Title/Abstract] OR "ce arom"[Title/Abstract] OR "ce limo"[Title/Abstract] OR "ce major"[Title/Abstract] OR "ce quin"[Title/Abstract] OR "ce quine"[Title/Abstract] OR "ce vi sol"[Title/Abstract] OR "ce vita"[Title/Abstract] OR "ce vitan"[Title/Abstract] OR "cebetate"[Title/Abstract] OR "cebicure"[Title/Abstract] OR "cebiolon"[Title/Abstract] OR "cebion"[Title/Abstract] OR "cebione"[Title/Abstract] OR "cecap"[Title/Abstract] OR "cecon"[Title/Abstract] OR "cecon drops"[Title/Abstract] OR "cecon solution"[Title/Abstract] OR "cecone"[Title/Abstract] OR "cecorbin"[Title/Abstract] OR "cecorbine"[Title/Abstract] OR "cecorbyl"[Title/Abstract] OR "cecorbyle"[Title/Abstract] OR "cecrisina"[Title/Abstract] OR "cedon"[Title/Abstract] OR "cedone"[Title/Abstract] OR "cedoxon"[Title/Abstract] OR "cedoxone"[Title/Abstract] OR "cee 500"[Title/Abstract] OR "ceevifil"[Title/Abstract] OR "cegiolan"[Title/Abstract] OR "celaskon"[Title/Abstract] OR "celaskone"[Title/Abstract] OR "celin"[Title/Abstract] OR "celine"[Title/Abstract] OR "cenetone"[Title/Abstract] OR "cenol"[Title/Abstract] OR "cenolate"[Title/Abstract] OR "cequinyl"[Title/Abstract] OR "cereon"[Title/Abstract] OR "cergona"[Title/Abstract] OR "cescorbat"[Title/Abstract] OR "cetamican"[Title/Abstract] OR "cetamid"[Title/Abstract] OR "cetamin knoll australia"[Title/Abstract] OR "cetamine"[Title/Abstract] OR "cetamine knoll australia"[Title/Abstract] OR "cetebe"[Title/Abstract] OR "ceterapion"[Title/Abstract] OR "ceterapione"[Title/Abstract] OR "cetrinets"[Title/Abstract] OR "cevalin"[Title/Abstract] OR "cevaline"[Title/Abstract] OR "cevatine"[Title/Abstract] OR "cevex"[Title/Abstract] OR "cevibram"[Title/Abstract] OR "cevigal"[Title/Abstract] OR "cevigen"[Title/Abstract] OR "cevigol"[Title/Abstract] OR "cevikap"[Title/Abstract] OR "cevilat"[Title/Abstract] OR "cevimin"[Title/Abstract] OR "cevimine"[Title/Abstract] OR "cevisol"[Title/Abstract] OR "cevit"[Title/Abstract] OR "cevita"[Title/Abstract] OR "cevitamic acid"[Title/Abstract] OR "cevitamin"[Title/Abstract] OR "cevitaminic acid"[Title/Abstract] OR "cevitaminum kolin"[Title/Abstract] OR "cevitan"[Title/Abstract] OR "cevite"[Title/Abstract] OR "cevitex"[Title/Abstract] OR "cevitil"[Title/Abstract] OR "cevitol"[Title/Abstract] OR "cewin"[Title/Abstract] OR "chewcee"[Title/Abstract] OR "chivibit c"[Title/Abstract] OR "ci drol"[Title/Abstract] OR "ciamin"[Title/Abstract] OR "ciamina ormo"[Title/Abstract] OR "ciergin"[Title/Abstract] OR "cifilina"[Title/Abstract] OR "cipca"[Title/Abstract] OR "cisir"[Title/Abstract] OR "citamino"[Title/Abstract] OR "cith"[Title/Abstract] OR "citoascorbina"[Title/Abstract] OR "citoxyl"[Title/Abstract] OR "citran"[Title/Abstract] OR "citravite"[Title/Abstract] OR "citritabs"[Title/Abstract] OR "citrovitamina"[Title/Abstract] OR "civigor"[Title/Abstract] OR "civitin"[Title/Abstract] OR "civitine"[Title/Abstract] OR "co biagini"[Title/Abstract] OR "concemin"[Title/Abstract] OR "cortalex"[Title/Abstract] OR "d ascorbic acid"[Title/Abstract] OR "d xyloascorbic acid"[Title/Abstract] OR "dagrascorbin"[Title/Abstract] OR "dagravit c"[Title/Abstract] OR "dancimin c"[Title/Abstract] OR "davitamon c"[Title/Abstract] OR "dayvital"[Title/Abstract] OR "delo c"[Title/Abstract] OR "difvitamin c"[Title/Abstract] OR "dropice"[Title/Abstract] OR "dumovit c"[Title/Abstract] OR "dumovite c"[Title/Abstract] OR "e ascorbic acid"[Title/Abstract] OR "e xyloascorbic acid"[Title/Abstract] OR "erftamin c"[Title/Abstract] OR "erftamine c"[Title/Abstract] OR "esuron"[Title/Abstract] OR "esurvit"[Title/Abstract] OR "esurvite"[Title/Abstract] OR "Ferrous Ascorbate"[Title/Abstract] OR "flavettes"[Title/Abstract] OR "godabion c"[Title/Abstract] OR "gregovite c"[Title/Abstract] OR "hicee"[Title/Abstract] OR "Hybrin"[Title/Abstract] OR "ido c"[Title/Abstract] OR "ikacee"[Title/Abstract] OR "inovitan c"[Title/Abstract] OR "irocevit"[Title/Abstract] OR "irocevite"[Title/Abstract] OR "jarexin"[Title/Abstract] OR "jarexine"[Title/Abstract] OR "l 3 keto hexuronic acid lactone"[Title/Abstract] OR "L Ascorbic Acid"[Title/Abstract] OR "l xylo ascorbic acid"[Title/Abstract] OR "l xyloascorbic acid"[Title/Abstract] OR "lacivit"[Title/Abstract] OR "lacivite"[Title/Abstract] OR "laroscorbine"[Title/Abstract] OR "leder c"[Title/Abstract] OR "lemascorb"[Title/Abstract] OR "levo ascorbate"[Title/Abstract] OR "levo ascorbic acid"[Title/Abstract] OR "limcee"[Title/Abstract] OR "limo ce"[Title/Abstract] OR "liqui cee"[Title/Abstract] OR "Magnesium Ascorbate"[Title/Abstract] OR "Magnesium Ascorbicum"[Title/Abstract] OR "Magnesium di L Ascorbate"[Title/Abstract] OR "Magnorbin"[Title/Abstract] OR "mega c/a plus"[Title/Abstract] OR "myascorbin"[Title/Abstract] OR "natrascorb"[Title/Abstract] OR "novo ascorbic"[Title/Abstract] OR "nybadol"[Title/Abstract] OR "paa 500"[Title/Abstract] OR "parkovit c"[Title/Abstract] OR "pascorbin"[Title/Abstract] OR "pharmascorbine"[Title/Abstract] OR "pharmatovit c"[Title/Abstract] OR "pharmatovite c"[Title/Abstract] OR "planavit c"[Title/Abstract] OR "planavite c"[Title/Abstract] OR "plivit c"[Title/Abstract] OR "plivite c"[Title/Abstract] OR "potassium ascorbate"[Title/Abstract] OR "pro c"[Title/Abstract] OR "proscorbin"[Title/Abstract] OR "proscorbine"[Title/Abstract] OR "redoxon"[Title/Abstract] OR "redoxon c"[Title/Abstract] OR "redoxon forte"[Title/Abstract] OR "ribena"[Title/Abstract] OR "scorbacid"[Title/Abstract] OR "scorbacide"[Title/Abstract] OR "scorbettes"[Title/Abstract] OR "scorbex"[Title/Abstract] OR "scorbin c"[Title/Abstract] OR "scorbitol"[Title/Abstract] OR "scorbumine"[Title/Abstract] OR "scottavit c"[Title/Abstract] OR "scottavite c"[Title/Abstract] OR "secorbate"[Title/Abstract] OR "sevalin"[Title/Abstract] OR "sigmavit c"[Title/Abstract] OR "sigmavite c"[Title/Abstract] OR "sodascorbate"[Title/Abstract] OR "Sodium Ascorbate"[Title/Abstract] OR "sodium l ascorbate"[Title/Abstract] OR "sweetcee"[Title/Abstract] OR "synum c"[Title/Abstract] OR "take c"[Title/Abstract] OR "tanvimil c"[Title/Abstract] OR "testascorbic"[Title/Abstract] OR "ucemine c"[Title/Abstract] OR "upsa c"[Title/Abstract] OR "upsavit"[Title/Abstract] OR "vagi c"[Title/Abstract] OR "vi c 500"[Title/Abstract] OR "vi ci sin"[Title/Abstract] OR "vi dom c"[Title/Abstract] OR "vicef"[Title/Abstract] OR "vicelat"[Title/Abstract] OR "vicetrin"[Title/Abstract] OR "vici monico"[Title/Abstract] OR "viciman"[Title/Abstract] OR "vicin"[Title/Abstract] OR "vicitina"[Title/Abstract] OR "vicon"[Title/Abstract] OR "viforcit"[Title/Abstract] OR "viforcite"[Title/Abstract] OR "viscorin"[Title/Abstract] OR "viscorine"[Title/Abstract] OR "vita cedol orange"[Title/Abstract] OR "vita gem c"[Title/Abstract] OR "vitac"[Title/Abstract] OR "vitace"[Title/Abstract] OR "vitacee"[Title/Abstract] OR "vitaci"[Title/Abstract] OR "vitacimin"[Title/Abstract] OR "vitacimine"[Title/Abstract] OR "vitacin"[Title/Abstract] OR "vitacine"[Title/Abstract] OR "Vitamin C"[Title/Abstract] OR "vitamin c 1"[Title/Abstract] OR "vitaplex c"[Title/Abstract] OR "vitapric"[Title/Abstract] OR "vitapur c"[Title/Abstract] OR "vitasan c"[Title/Abstract] OR "vitascorbin"[Title/Abstract] OR "vitascorbine"[Title/Abstract] OR "vitascorbol"[Title/Abstract] OR "vitascorbol 500"[Title/Abstract] OR "vitelix c"[Title/Abstract] OR "vitocee"[Title/Abstract] OR "vorange"[Title/Abstract] OR "wandervit c"[Title/Abstract] OR "wandervite c"[Title/Abstract] OR "witamina c"[Title/Abstract] OR "xitix"[Title/Abstract] OR "xon ce"[Title/Abstract] OR "xyloascorbic acid"[Title/Abstract] |  |  | "acid ascorbic"[Title/Abstract] OR "acid l ascorbic"[Title/Abstract] OR "acidum ascorbicum"[Title/Abstract] OR "adenex"[Title/Abstract] OR "allercorb"[Title/Abstract] OR "antiscorbutic vitamin"[Title/Abstract] OR "ascor"[Title/Abstract] OR "ascorbate"[Title/Abstract] OR "ascorbate sodium"[Title/Abstract] OR "Ascorbic Acid"[Title/Abstract] OR "ascorbicap"[Title/Abstract] OR "ascorbico"[Title/Abstract] OR "ascorbin"[Title/Abstract] OR "ascorbinic acid"[Title/Abstract] OR "ascorbit"[Title/Abstract] OR "ascorbone"[Title/Abstract] OR "ascorbutina"[Title/Abstract] OR "ascorbyl"[Title/Abstract] OR "ascorin"[Title/Abstract] OR "ascorteal"[Title/Abstract] OR "ascorvit"[Title/Abstract] OR "c level"[Title/Abstract] OR "c long"[Title/Abstract] OR "c sol"[Title/Abstract] OR "c tamin"[Title/Abstract] OR "c tonic"[Title/Abstract] OR "c vimin"[Title/Abstract] OR "c vit"[Title/Abstract] OR "c vita"[Title/Abstract] OR "c vital"[Title/Abstract] OR "c will"[Title/Abstract] OR "cantan"[Title/Abstract] OR "cantaxin"[Title/Abstract] OR "catavin c"[Title/Abstract] OR "ce vi sol"[Title/Abstract] OR "cebicure"[Title/Abstract] OR "cebion"[Title/Abstract] OR "cebione"[Title/Abstract] OR "cecap"[Title/Abstract] OR "cecon"[Title/Abstract] OR "cee 500"[Title/Abstract] OR "cegiolan"[Title/Abstract] OR "celaskon"[Title/Abstract] OR "celin"[Title/Abstract] OR "celine"[Title/Abstract] OR "cenetone"[Title/Abstract] OR "cenolate"[Title/Abstract] OR "cereon"[Title/Abstract] OR "cergona"[Title/Abstract] OR "cescorbat"[Title/Abstract] OR "cetamid"[Title/Abstract] OR "cetamine"[Title/Abstract] OR "cetebe"[Title/Abstract] OR "cevalin"[Title/Abstract] OR "cevaline"[Title/Abstract] OR "cevatine"[Title/Abstract] OR "cevex"[Title/Abstract] OR "cevimin"[Title/Abstract] OR "cevisol"[Title/Abstract] OR "cevit"[Title/Abstract] OR "cevitamic acid"[Title/Abstract] OR "cevitamin"[Title/Abstract] OR "cevitan"[Title/Abstract] OR "cevitex"[Title/Abstract] OR "cewin"[Title/Abstract] OR "ciamin"[Title/Abstract] OR "cipca"[Title/Abstract] OR "cisir"[Title/Abstract] OR "citamino"[Title/Abstract] OR "cith"[Title/Abstract] OR "citran"[Title/Abstract] OR "concemin"[Title/Abstract] OR "cortalex"[Title/Abstract] OR "d ascorbic acid"[Title/Abstract] OR "davitamon c"[Title/Abstract] OR "Ferrous Ascorbate"[Title/Abstract] OR "hicee"[Title/Abstract] OR "Hybrin"[Title/Abstract] OR "ido c"[Title/Abstract] OR "L Ascorbic Acid"[Title/Abstract] OR "l xyloascorbic acid"[Title/Abstract] OR "laroscorbine"[Title/Abstract] OR "lemascorb"[Title/Abstract] OR "levo ascorbic acid"[Title/Abstract] OR "liqui cee"[Title/Abstract] OR "Magnesium Ascorbate"[Title/Abstract] OR "natrascorb"[Title/Abstract] OR "pascorbin"[Title/Abstract] OR "planavit c"[Title/Abstract] OR "potassium ascorbate"[Title/Abstract] OR "pro c"[Title/Abstract] OR "proscorbin"[Title/Abstract] OR "redoxon"[Title/Abstract] OR "ribena"[Title/Abstract] OR "scorbacid"[Title/Abstract] OR "secorbate"[Title/Abstract] OR "Sodium Ascorbate"[Title/Abstract] OR "sodium l ascorbate"[Title/Abstract] OR "testascorbic"[Title/Abstract] OR "vagi c"[Title/Abstract] OR "vicelat"[Title/Abstract] OR "vicin"[Title/Abstract] OR "vicon"[Title/Abstract] OR "viforcit"[Title/Abstract] OR "viscorin"[Title/Abstract] OR "vitac"[Title/Abstract] OR "vitace"[Title/Abstract] OR "vitacee"[Title/Abstract] OR "vitacimin"[Title/Abstract] OR "vitacin"[Title/Abstract] OR "Vitamin C"[Title/Abstract] OR "vitamin c 1"[Title/Abstract] OR "vitascorbol"[Title/Abstract] OR "witamina c"[Title/Abstract] OR "xitix"[Title/Abstract] OR "xyloascorbic acid"[Title/Abstract] | 80,844 | 9:39:13 |

**WOS search-history**

| Entitlements | # | Search Query | Database | Results | Date Run |
| --- | --- | --- | --- | --- | --- |
| - WOS: 1900 to 2023 - CSCD: 1989 to 2023 - DIIDW: 1966 to 2023 - KJD: 1980 to 2023 - MEDLINE: 1950 to 2023 - PPRN: 1991 to 2023 - SCIELO: 2002 to 2023 | 91 | ((((((((((((((((((TS=(Acid, Ascorbic)) OR TS=(Acid, L-Ascorbic)) OR TS=(Ascorbate, Ferrous)) OR TS=(Ascorbate, Magnesium)) OR TS=(Ascorbate, Sodium)) OR TS=(Ascorbic Acid)) OR TS=(Ascorbic Acid, Monosodium Salt)) OR TS=(di-L-Ascorbate, Magnesium)) OR TS=(Ferrous Ascorbate)) OR TS=(Hybrin)) OR TS=(L Ascorbic Acid)) OR TS=(L-Ascorbic Acid)) OR TS=(Magnesium Ascorbate)) OR TS=(Magnesium Ascorbicum)) OR TS=(Magnesium di L Ascorbate)) OR TS=(Magnesium di-L-Ascorbate)) OR TS=(Magnorbin)) OR TS=(Sodium Ascorbate)) OR TS=(Vitamin C) and Preprint Citation Index (Exclude – Database) | All Databases | 373735 | Sat Apr 08 2023 23:10:59 GMT+0800 (中国标准时间) |
| - WOS: 1900 to 2023 - CSCD: 1989 to 2023 - DIIDW: 1966 to 2023 - KJD: 1980 to 2023 - MEDLINE: 1950 to 2023 - PPRN: 1991 to 2023 - SCIELO: 2002 to 2023 | 92 | (((TS=(Pregnant Women)) OR TS=(Pregnant Woman)) OR TS=(Woman, Pregnant)) OR TS=(Women, Pregnant) and Preprint Citation Index (Exclude – Database) | All Databases | 226425 | Sat Apr 08 2023 23:12:29 GMT+0800 (中国标准时间) |
| - WOS: 1900 to 2023 - CSCD: 1989 to 2023 - DIIDW: 1966 to 2023 - KJD: 1980 to 2023 - MEDLINE: 1950 to 2023 - PPRN: 1991 to 2023 - SCIELO: 2002 to 2023 | 93 | #91 AND #92 and Preprint Citation Index (Exclude – Database) | All Databases | 2887 | Sat Apr 08 2023 23:14:07 GMT+0800 (中国标准时间) |
